# Standardized community management on the diagnosis, treatment, and risk factors control for non-valvular atrial fibrillation in elderly patients

**DOI:** 10.1186/s12875-023-02207-1

**Published:** 2023-11-30

**Authors:** Wei Wang, Yufeng Gu, Shan Wei, Juan Xie, Xiuli Zheng, Yan Yu

**Affiliations:** 1 Deparment of General practice, Zhuanqiao Community Health Service Center, Shanghai, Minhang District China; 2 Department of Infectious Diseases, The Fifth People’s Hospital of Shanghai, Shanghai, China; 3 Deparment of General practice, The Fifth People’s Hospital of Shanghai, Shanghai, China; 4 Department of Pharmacy, The Fifth People’s Hospital of Shanghai, Shanghai, China

**Keywords:** Atrial fibrillation, Elderly, Community hospital, Health management, Risk factors

## Abstract

**Background:**

By investigating the knowledge, medication, occurrence of complications, and risks among elderly non-valvular atrial fibrillation (NVAF) patients in Shanghai communities, and providing standardized comprehensive management and follow-up, we aim to explore the impact of standardized community management on improving disease awareness, standardizing atrial fibrillation (AF) treatment, reducing the risk of complications occurrence, and addressing risk factors for AF patients.

**Methods:**

This research selected elderly atrial fibrillation patients from Zhuanqiao Community Health Service Center, Minhang District, Shanghai from July 2020 to October 2022. Their personal health records and examination results were reviewed, and the incidence of AF, awareness, medication, and complications were investigated. Age-adjusted Charlson Comorbidity Index (aCCI), CHA_2_DS_2_-VASc score, and HAS-BLED score were used to evaluate disease burden, thromboembolic risk, and bleeding risk, respectively. The patients were subjected to standardized community management, and the compliance rate of disease awareness, treatment, resting heart rate, blood pressure, fasting blood glucose, and body mass index (BMI) were assessed at the baseline, 6 months and 1 year after management.

**Results:**

A total of 243 NVAF patients were included, with an average aCCI score of (4.5 ± 1.1). Among them, 28% of the patients were aware of their AF, and 18.1% of the patients were aware of the hazards of AF. Of the patients, 11.9% used anticoagulant drugs, including 6.6% and 5.3% for warfarin and non-vitamin K antagonist oral anticoagulants (NOACs), respectively. 7% of patients used antiplatelet drugs. 26.7% of the patients used heart rate control drugs. 10.3% of the patients experienced thromboembolic events, and 0.8% of the patients experienced bleeding events. 93.0% of the patients were at high risk of thromboembolism, and 24.7% of the patients were at high risk of bleeding. Compared with the baseline, there were significant statistical differences (*P* < 0.001) in disease awareness, awareness of the hazards of AF, use of anticoagulant drugs and heart rate control drugs, and control of risk factors among NVAF patients after standardized community management. Moreover, with the extension of management time, there was a linear increase in the awareness of NVAF, awareness of the hazards of AF, utilization rate of anticoagulant drugs, utilization rate of heart rate control drugs, blood pressure, blood glucose, and BMI compliance rate (*P* < 0.001).

**Conclusion:**

Currently, the awareness, treatment, and control of risk factors for AF in elderly NVAF patients in Shanghai community are not satisfactory. Standardized community management helps to improve the diagnosis, treatment, and control of risk factors in AF.

## Background

Atrial fibrillation (AF), is a common type of rapid heart rhythm disorder. As of 2019, there were approximately 59.7 million AF patients worldwide [1]. The age-adjusted prevalence rate of AF in China is 0.74%, with respective rates of 1.83% and 1.92% in males and females in the 60-year-old population [2]. AF patients have double the hospitalization rate compared to non-AF patients, with approximately 30% of AF patients being hospitalized at least once per year [3], leading to a 1.5 to 2-fold increase in overall mortality [4], which greatly increases the economic and medical burden on families and society. Anticoagulation and rhythm control treatment are important measures for reducing the risk of AF complications, and comprehensive management of upstream treatment and underlying conditions is also crucial [5, 6]. However, due to limited patient understanding of AF and its hazards, inadequate awareness of AF anticoagulation treatment among community doctors, and the inconvenience of long-term standardized use of anticoagulant medications, most AF patients in China do not receive standardized treatment, especially anticoagulation therapy. Research has found that the anticoagulation rate among AF patients in the community is only 24% [7]. Therefore, the standardization of AF treatment and management to prevent complications has become one of the urgent clinical problems to be addressed. With the promotion of the hierarchical diagnosis and treatment model, community hospitals have become the main battlefield for chronic disease management, and standardized management models for chronic diseases such as hypertension, diabetes, and cardiovascular diseases have gradually improved [8–14]. However, the current community management of AF patients is not yet standardized, and most of them only involve warfarin anticoagulation treatment and management [15], with limited research on comprehensive management [16, 17]. Based on the current situation of high incidence, low awareness, non-standardized treatment, and high hazards of AF in the elderly population, this study included non-valvular atrial fibrillation (NVAF) patients over 60 years old in our hospital. Based on the existing personal health records, combined with other basic chronic disease information, the AF information records were improved, and their understanding of AF, disease management, complications, and risks were investigated. Standardized comprehensive management and follow-up were conducted to explore the effects of standardized community management of AF on improving disease awareness, standardizing AF treatment, reducing the risk of complications, and risk factors for AF patients.

## Methods

### Study subjects

**Fig. 1:**
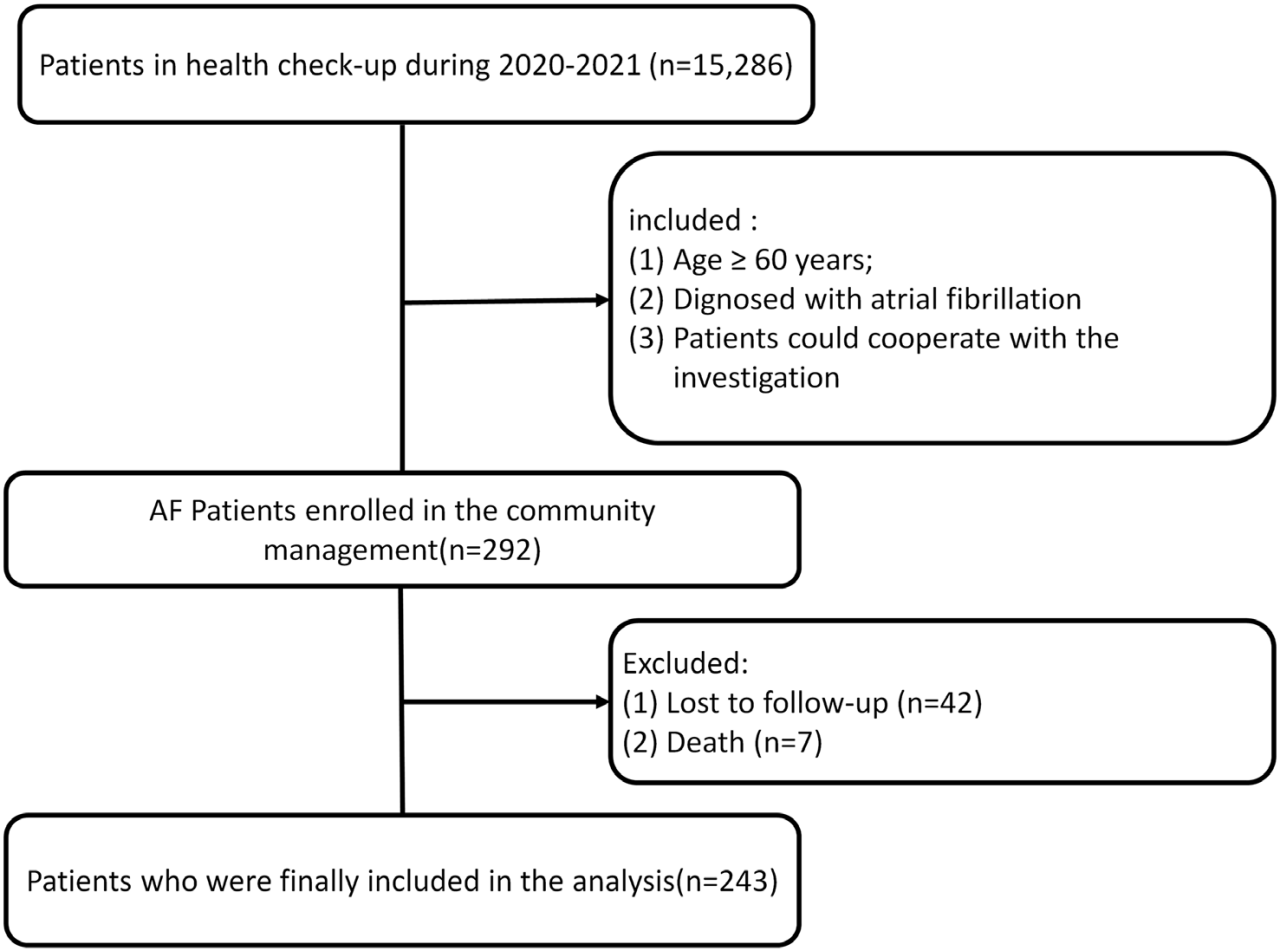
Flow diagram of the pilot study

Elderly AF patients over 60 years old were screened from the physical examination center of our hospital from July 2020 to October 2022 and followed up for one year months (Fig. 1). This single-arm, pre-post design study was part of the program (ChiCTR2000033145), which received ethics approval (2,018,173) from the Institutional Review Board of Fifth People’s Hospital. All patients provided written informed consent before enrolling in the study. The inclusion criteria were as follows: (1) age ≥ 60 years; (2) diagnosed with AF by electrocardiography or 24-hour dynamic electrocardiography, lasting > 30 s. The exclusion criteria were as follows: (1) AF caused by hyperthyroidism; (2) valvular AF, including AF in patients with mechanical valve replacement or moderate to severe mitral valve stenosis; (3) patients with mental illness, cognitive dysfunction, severe audiovisual dysfunction, or who could not cooperate with the investigation and follow-up for unforeseen reasons.

### Data collection

Before conducting the study, uniform training was provided to all healthcare personnel involved in the study to ensure consistency. Personal health records and physical examination results of the included patients were reviewed, and their history of AF, knowledge of AF, medication use, and occurrence of complications were investigated. The following relevant data were collected and evaluated: (1) gender, age, height, weight, smoking and alcohol consumption, ventricular rate, blood pressure, and blood glucose level; (2) comorbidities and disease burden: age-adjusted Charlson Comorbidity Index (aCCI) [11]; (3) AF-related information: occurrence of symptoms (palpitations, fatigue, chest tightness, decreased exercise tolerance, dyspnea after exertion), awareness of the disease, methods of discovery, disease duration, awareness of hazards (stroke and thromboembolism, heart failure, myocardial infarction, cognitive decline, dementia, renal impairment, decreased quality of life, awareness of one or more hazards is considered awareness, otherwise unaware), anticoagulation treatment and rhythm control medication use, and occurrence of complications; (4) assessment of the risk of AF complications: CHA_2_DS_2_-VASc score: congestive heart failure, hypertension, age ≥ 65 years, diabetes, vascular diseases, and female gender each score 1 point, age (A2) ≥ 75 years score 2 points, and stroke/transient ischemic attack/thromboembolism and age ≥ 75 years each score 2 points. A score ≥ 2 for males and ≥ 3 for females indicates a high risk of stroke and requires long-term anticoagulation treatment. HAS-BLED bleeding risk score: systolic blood pressure levels higher than 160 mmHg, abnormal liver or renal function, stroke, bleeding, labile international normalized ratio (INR), age > 65 years each score 1 point, and drug or alcohol use each score 1 or 2 points. A score ≥ 3 indicates a high risk of bleeding but is not a contraindication for anticoagulation treatment. Instead, it serves as a reminder to strengthen monitoring during anticoagulation and screen for and correct reversible factors increasing the risk of bleeding; (5) management of AF symptoms and risk factors: the following indicators were followed up before and after management at 6 months and 1 year, and meeting the following target values was considered satisfactory: ① resting heart rate < 80 beats/min; ② blood pressure < 130/80 mmHg for patients with hypertension; ③ fasting blood glucose < 7.0 mmol/L for patients with diabetes; ④ body mass index (BMI) < 24 kg/m^2^.

### Standardized community management for AF

**Fig. 2:**
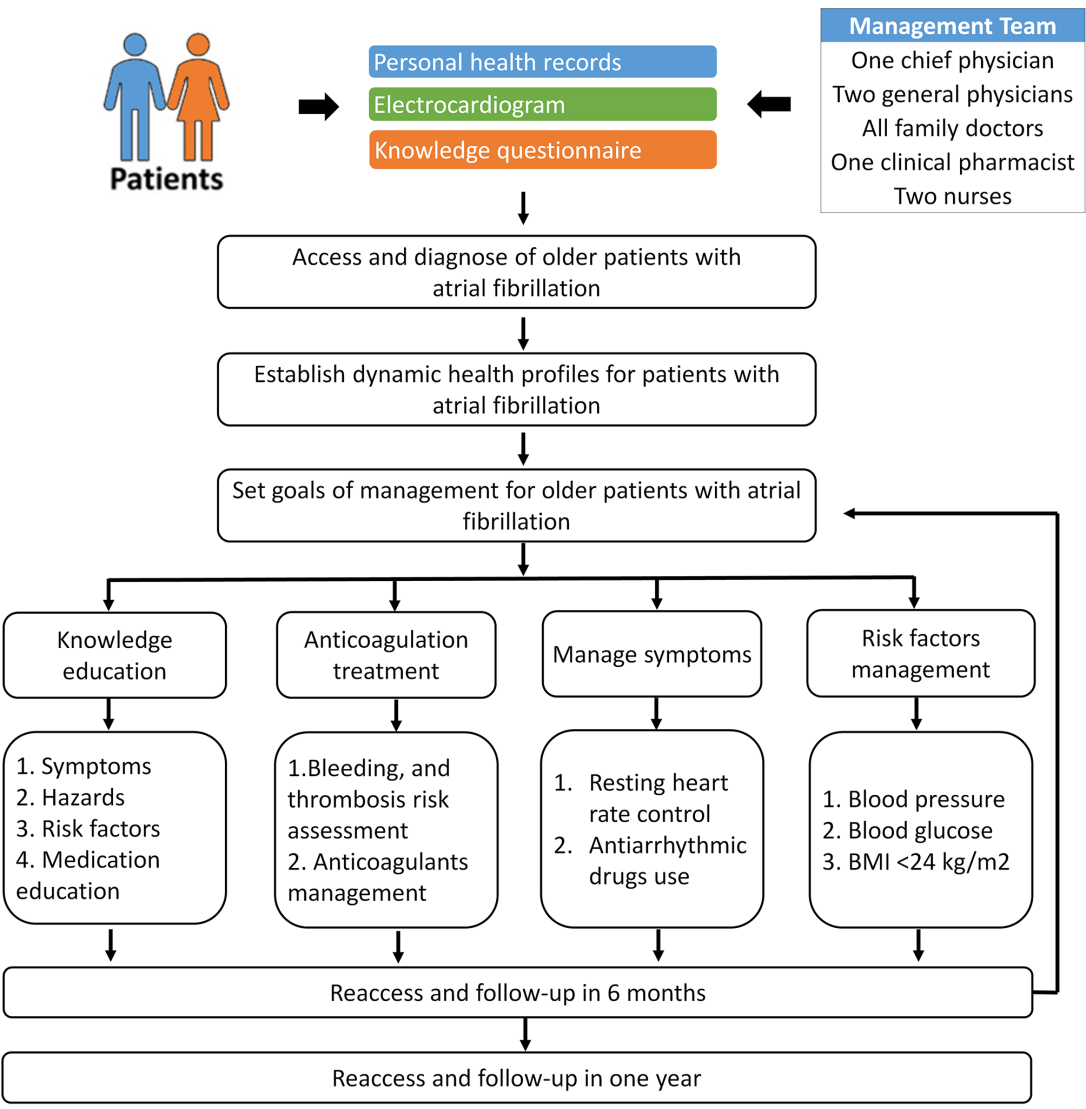
Standardized community management program on non-valvular atrial fibrillation in elderly patients

First, the director from the Cardiovascular Department of the Fifth People’s Hospital provided professional training to our general practitioners, including all family doctors, and helped to establish a standardized management team for AF. The team consisted of one specialist director, two experienced general practitioners who had received training, all family doctors, one clinical pharmacist, and two nurses. A specialized clinic for AF was set up, with the experienced general practitioners in charge. The clinic carried out a combination of individual and group health assessments and education. On one hand, every AF patient participating in the study were contacted regularly by phone and asked to come to our specialized clinic for evaluation of their current condition, timely adjustment of diagnosis and treatment plans, and personalized health education. This improved to refresh the dynamic health records of AF patients. For patients who were unable to visit the hospital due to various reasons, assessments and health education were conducted through means such as phone calls, WeChat, and home visits by family doctors. On the other hand, occasional expert lectures, online promotions, patient exchange meetings, and knowledge competitions were held for group health education activities. The management continued for 1 year, and all relevant patient information was collected during the follow-up visits in the the management period (As shown in Fig. 2).

### Statistic analysis

SPSS 25.0 was used for data statistical analysis. All normally distributed quantitative data was presented as mean ± standard deviation (mean ± SD), while qualitative data was presented as frequency and percentage. Chi-square test was used for comparing indicators before and after the management. *P* < 0.05 indicates statistical significance.

## Results

### Basic information and co-morbidity of NVAF patients

After excluding 42 cases lost to follow-up and 7 deaths, a total of 243 NVAF patients were included in this study (Table 1), with an average age of (73.6 ± 6.6) years. Among them, 97 cases (39.9%) were aged ≥ 75 years. The average aCCI score was (4.5 ± 1.1), with 105 cases (43.2%) of patients having hypertension, 47 cases (19.3%) having diabetes, and 14 cases (5.8%) having heart failure.

**Table 1:** The characteristics and comorbidities of 243 NVAF patients

Characteristics	Value
**Age(years)**	73.6 ± 6.6
60–64	12(4.9)
65–74	134(55.1)
≥ 75	97(39.9)
Male	150(61.7)
**Anthropometric data**	
BMI(kg/m^2^)	25.9 ± 3.6
<24	76(31.3)
24 ~ 28	106(43.6)
>28	61(25.1)
Current Smoking	22(9.1)
Current Drinking	27(11.1)
**Medical history**	
Heart Failure	14(5.8)
Hypertension	105(43.2)
Diabetes mellitus	47(19.3)
Vascular disease	17(7.0)
**Scores**	
aCCI	4.5 ± 1.1
CHA_2_DS_2_-VASc	3.1 ± 1.2
HAS-BLED	1.7 ± 1.0

The values are expressed as the mean ± standard deviation (SD) or n(%) of patients

NVAF: non-valvular atrial fibrillation; BMI: body mass index; Vascular disease: atherosclerosis, arteriosclerosis, peripheral artery disease, deep vein thrombosis and varicose veins; aCCI: age-adjusted charlson comorbidity index

### AF incidence, cognitive status, medication, complications, and risk in 243 NVAF patients

Among the 243 NVAF patients in Tables 2 and 229 cases (94.2%) had at least one AF symptom, but only 68 cases (28%) were aware of their AF condition, 64 cases (26.3%) discovered AF through means other than physical examination, 48 cases (19.8%) had an AF duration of more than 2 years, 44 cases (18.1%) were aware of the risks associated with AF, 29 cases (11.9%) used anticoagulant drugs, including warfarin, non-vitamin K antagonist oral anticoagulants (NOACs), with 16 cases (6.6%), 13 cases (5.3%) respectively, 65 cases (26.7%) used heart rate control drugs, 25 cases (10.3%) experienced thromboembolic events, and 2 cases (0.8%) experienced bleeding events. Among them, 226 cases (93.0%) were at high risk of stroke, and 60 cases (24.7%) were at high risk of bleeding.

**Table 2:** The occurrence, medication usage, complications and risk of AF in 243 patients

Characteristics	Value(%)
Atrial fibrillation symptoms, n (%)	229(94.2)
**Pathway of AF discovery, n (%)**	
Health checkup	179(73.7)
Others	64(26.3)
**Course of atrial fibrillation, n (%)**	
<2 years	195(80.2)
2 ~ 5 years	23(9.5)
>5 years	25(10.3)
Awareness of atrial fibrillation hazards, n(%)	44(18.1)
Use of anticoagulants, n(%)	29(11.9)
**Types of anticoagulants**, n(%)	
No	197(81.1)
Warfarin	16(6.6)
NOACs	13(5.3)
Use of antiplatelets	17(7.0)
Use of heart rate control drugs	65(26.7)
**CHA2DS2-VASc score, n (%)**	
High risk	226(93.0)
Low/middle risk	17(7.0)
**HAS-BLED score, n (%)**	
High	60(24.7)
Low/middle risk	183(75.3)
**Previous thromboembolic events, n (%)**	25(10.3)
With anticoagulants	3(1.2)
**Previous bleeding events, n (%)**	2(0.8)
With anticoagulants	0(0.0)

The values are expressed as n(%) of patients

Others: atrial fibrillation diagnosed in outpatient visits due to discomfort or identified and diagnosed during hospitalization for other illnesses in patients

### Community-based standardized management improves AF awareness, anticoagulation treatment and risk factors control in 243 NVAF patients

Compared with the baseline at the beginning of the study, the awareness of AF and the awareness of the risks associated with AF in NVAF patients under community-based standardized management showed statistically significant changes (*P* < 0.001). Moreover, as the duration of management increased, there was a linear increase in the AF awareness and AF risk awareness in NVAF patients (*P* < 0.001). In addition, the usage rates of anticoagulant drugs and heart rate control drugs in NVAF patients under community-based standardized management showed a linear increase and had statistical significance (*P* < 0.001). The rates of blood pressure, blood sugar, and BMI control in NVAF patients under community-based standardized management showed a linear increase and had statistical significance (*P* < 0.05), but no significant changes were observed in heart rate control (*P* = 0.363). See Table 3.

**Table 3:** Comparison of AF awareness, medication utilization, symptoms and risk factors before and after management in 243 NVAF patients

Characteristics	Baseline	6 months	1 year	*P* value
**Education, n(%)**				
Awareness of AF, n(%)	68(28.0)	178(73.3)	233(95.9)	<0.001
Awareness of AF hazards, n(%)	44(18.1)	177(72.8)	218(89.7)	<0.001
**Atrial fibrillation treatment, n(%)**				
Use of anticoagulants	29(11.9)	66(27.1)	122(50.3)	<0.001
Types of anticoagulants				
Warfarin	16(6.6)	21(8.6)	22(9.1)	0.565
NOACs	13(5.3)	45(18.5)	100(41.2)	<0.001
Use of antiplatelets	17(7.0)	18(7.4)	15(6.2)	0.860
Use of heart rate control drugs	65(26.7)	96(39.5)	114(46.9)	<0.001
**Compliance of symptoms and risk factors, n(%)**				
Heart rate reaches target	191(78.6)	199(81.9)	203(83.5)	0.363
Blood pressure reaches target	72(29.6)	92(37.9)	105(43.2)	0.008
Blood glucose reaches target	196(80.7)	209(86.0)	215(88.5)	0.047
BMI reaches target	76(31.3)	92(37.9)	102(42.0)	0.048
**Adverse events within follow-up, n(%)**				
New thromboembolic events	0(0.0)	/	4(1.6)	0.045
With anticoagulants	0(0.0)	/	0(0.0)	/
New bleeding events	0(0.0)	/	3(1.2)	0.082
With anticoagulants	0(0.0)	/	1(0.8)	0.625

## Discussion

This study investigated the awareness, medication use, occurrence of complications and risks in community-dwelling elderly patients with NVAF, and provided standardized comprehensive management. The study had a follow-up loss rate of 16.8%, which was within an acceptable range, and ultimately included 243 NVAF patients.

This study showed that as high as 94.2% of AF patients had experienced at least one symptom of AF, with approximately 36.6% of patients attributing their symptoms to aging. Among them, only 28% of patients were aware of their AF condition, and only 18.1% of patients were aware of the hazards associated with AF, suggesting insufficient awareness of AF disease among elderly AF patients. Furthermore, 26.3% of patients were diagnosed with AF through means other than routine check-ups, indicating that only 1.7% of patients became aware of their AF condition after this check-up. Despite participating in regular health check-ups and receiving written reports, a large portion of elderly individuals were unaware of their AF condition. These findings suggest that community-based family doctor teams should strengthen the interpretation of health check-up results for elderly individuals in their jurisdiction, and enhance health education for AF patients, including knowledge about AF, treatment measures and their importance, as well as the necessity of regular monitoring and follow-ups.

Elderly patients often have a high prevalence of chronic comorbidities, such as hypertension, diabetes, and coronary heart disease. The aCCI quantifies disease burden by considering the number, severity, and age of comorbidities in elderly patients, with > 3 points indicating high disease burden. Some studies have that when the aCCI score is > 3, the 10-year survival rate is ≤ 77.5% [18]. In this study, approximately 81.9% of NVAF patients had an aCCI score > 3, with an average aCCI score of 4.49 ± 1.07, suggesting a heavy burden of chronic diseases among elderly NVAF patients. Among them, approximately 43.2% had hypertension, 9.3% had diabetes, and 5.8% had heart failure. This is consistent with the results of the study conducted by Jiang J et al. [16]. Previous studies have found that AF, heart failure, hypertension, and coronary heart disease increase the risk of stroke by more than 5 times, 4 times, 3 times, and 1 time, respectively [19]. Therefore, it is necessary to strengthen anticoagulant treatment for AF patients with comorbidities of hypertension and heart failure, as well as control risk factors such as hypertension, heart failure, and coronary heart disease [20]. According to the CHA_2_DS_2_-VASc score, approximately 93.0% of patients in this study were considered high-risk for stroke and should receive long-term anticoagulant treatment. The rate of anticoagulant use in this study population was 11.9%, which was similar with a mere 11.2% of patients with AF in China on oral anticoagulants therapy according to the RE-LY study [21], but lower than that reported in other communities (24% [7] or 38% [16]), and much lower than the rates in Eastern European countries (40%) and North America (65.7%) [21]. Therefore, efforts should continue to be made to enhance anticoagulant treatment for AF patients.

This innovative study implemented community-based standardized comprehensive management for elderly AF patients. After 6 months of management, the anticoagulation rate of elderly NVAF patients increased to 34.6%, similar to elderly NVAF patients in other communities (38%) [16], and after 1 year of management, the anticoagulation rate (56.4%) approached that of Eastern Europe (40%) and North America (65.7%) [21]. Studies have shown that standard-dose NOACs in Asian populations can significantly reduce the risk of stroke or systemic embolism while reducing the risk of bleeding, demonstrating good efficacy and safety [22]. Therefore, patients taking NOACs have better medication compliance and lower discontinuation rates [23]. Currently, NOACs are easy to purchase and affordable in the community, which is also a reason for the improvement in anticoagulation rate and NOAC usage rate among AF patients.

In addition to anticoagulation and rhythm control treatments, active management of risk factors may reduce the risk of new-onset AF and improve the success rate of AF treatment [5]. Hypertension, diabetes, and obesity are common risk factors for AF, which also increase the risk of AF occurrence and the occurrence of AF complications, especially stroke, heart failure, and bleeding risks [24–26]. In existing studies, the incidence rate of HF in persistent AF is 44% [27], the annual incidence rate of ischemic stroke is about 2% [28], and the annual incidence rate of myocardial infarction ranges from 0.4 to 2.5% [29]. Therefore, the standardized community management strengthened the management of risk factors in AF patients. Results of the study also showed that after standardized community management, there was a linear increasing trend and statistical significance in the rate of blood pressure, blood sugar, and BMI control for NVAF patients. In future studies, researchers will continue to follow up to understand the impact of standardized management on the occurrence rate of complications, hospitalization rate, and mortality rate of AF patients.

This study has certain limitations. Firstly, the current management model is not yet perfect, and due to the impact of the COVID-19 pandemic, many offline activities have been replaced by online activities. In future research and promotion, it is necessary to further enrich the content of dynamic health records, standardize management details, strengthen the implementation of offline management activities, and refine follow-up indicators. Secondly, this study is a single-center small-sample study, and the samples are only sourced from elderly NVAF patients who participated in community health examinations, thus having certain limitations. In future research, multicenter studies can be conducted, and the sample size can be expanded to facilitate universal promotion of standardized management models. Finally, this study only followed up for one year, and the follow-up time was short, so it is unable to determine the impact of standardized management on the occurrence of complications in AF patients, improvement in quality of life, and long-term prognosis.

## Conclusions

The awareness of elderly NVAF patients in Shanghai communities about their own AF disease and the risks of AF is low, and the treatment situation for AF is not ideal. After standardized community management, the understanding, treatment, and management of risk factors for elderly NVAF patients have significantly improved. In the future, it is necessary to further improve a more standardized community hospital AF management model and explore the relationship between standardized management of AF and long-term prognosis.

## Data Availability

Data will be available upon reasonable request.
